# Geo-referenced population-specific microsatellite data across American continents, the MacroPopGen Database

**DOI:** 10.1038/s41597-019-0024-7

**Published:** 2019-04-03

**Authors:** Elizabeth R. Lawrence, Javiera N. Benavente, Jean-Michel Matte, Kia Marin, Zachery R. R. Wells, Thaïs A. Bernos, Nia Krasteva, Andrew Habrich, Gabrielle A. Nessel, Ramela Arax Koumrouyan, Dylan J. Fraser

**Affiliations:** 1 Department of Biology, Concordia University, 7141 Sherbrooke Street W., Montreal, Quebec H4B 1R6 Canada; 2 School of Environment, University of Auckland, PO Box 92019, Auckland, 1142 New Zealand; 3 Golder Associates, 7250, rue du Mile End, 3e étage, Montréal, Québec H2R 3A4 Canada; 4 BT Engineering Inc., 100 Craig Henry Drive, Suite 201, Nepean, Ontario K2G 5W3 Canada; 5 Department of Biology and Centre for Forest-Interdisciplinary Research, University of Winnipeg, Winnipeg, Manitoba R3B 2E9 Canada

**Keywords:** Macroecology, Biodiversity

## Abstract

Population genetic data from nuclear DNA has yet to be synthesized to allow broad scale comparisons of intraspecific diversity versus species diversity. The MacroPopGen database collates and geo-references vertebrate population genetic data across the Americas from 1,308 nuclear microsatellite DNA studies, 897 species, and 9,090 genetically distinct populations where genetic differentiation (F_ST_) was measured. Caribbean populations were particularly distinguished from North, Central, and South American populations, in having higher differentiation (F_ST_ = 0.12 vs. 0.07–0.09) and lower mean numbers of alleles (MNA = 4.11 vs. 4.84–5.54). While mammalian populations had lower MNA (4.86) than anadromous fish, reptiles, amphibians, freshwater fish, and birds (5.34–7.81), mean heterozygosity was largely similar across groups (0.57–0.63). Mean F_ST_ was consistently lowest in anadromous fishes (0.06) and birds (0.05) relative to all other groups (0.09–0.11). Significant differences in Family/Genera variance among continental regions or taxonomic groups were also observed. MacroPopGen can be used in many future applications including latitudinal analyses, spatial analyses (e.g. central-margin), taxonomic comparisons, regional assessments of anthropogenic impacts on biodiversity, and conservation of wild populations.

## Abstract (summary)

**Table:** 

Design Type(s)	species comparison design • individual genetic characteristics comparison design • data integration objective
Measurement Type(s)	genetic variation
Technology Type(s)	digital curation
Factor Type(s)	Taxon • Species
Sample Characteristic(s)	population of organisms

Machine-accessible metadata file describing the reported data (ISA-Tab format)

## Background and Summary

Collating large quantities of data is useful not only for assessing large-scale patterns but also for testing theories, informing conservation initiatives, and providing a valuable resource for future data comparisons. In particular, macro-ecological biodiversity assessments are becoming increasingly popular to identify hotspots of species biodiversity that can inform local management strategies^1–5^. However, populations, not species, are generally recognized as the appropriate scale for the management of sustainable harvesting and protection in endangered species legislation^6–8^. Nevertheless, population diversity – the number of genetically distinct populations within species – is typically excluded from most biodiversity syntheses and large-scale conservation planning (e.g.^1,9–13^). This has consequences when assessing biodiversity loss, as population extinction occurs at a much faster rate than species loss, and as such, a species’ vulnerability could be grossly misrepresented^14^.

Molecular markers provide an increasingly effective way to differentiate populations and estimate population diversity^15^. One example is the global population diversity estimate based on allozymes and restriction fragment length polymorphisms where authors found on average 220 populations per species and estimated annual loss of 16 million populations, a coarse estimate obtained by dividing the number of sampling locations by the sampling area^10^. The collated data from this study was not made publicly available for future usage and is outdated following the advancement of genetic tools. No study has formally revisited these concepts since this 1997^10^ study (^16–18^, but see^14,19,20^ for exceptions), indicating the need for collating population information.

Population genetic technologies have seen advances in recent years, switching from allozymes to microsatellites to single nucleotide polymorphisms (SNPs), largely due to the better resolution of within-population variation that more recent technologies provide^15,21^. Population structure studies and vulnerability assessments have used microsatellites as their molecular marker for the past two decades, yet this wealth of data has not been thoroughly collated, although a few authors have collated related information in the form of microsatellite genetic variation^9,12^, population density estimates^11^, and pairwise F_ST_ estimates^13^. Despite the great degree of data collation across these studies, no work has combined the geo-referencing of population-specific genetic variation, F_ST_ measurements, and the number of populations within a species to create a single database across a wide variety of taxa and geographic regions.

Here we provide the first description of the release of the Macro-ecological, Population Genetics Database (MacroPopGen Database) – a database that contains geo-referenced population-specific characteristics based on nuclear DNA microsatellites. It contains information on 897 species from 1,308 studies published between 1994–2017, and 9,090 distinct populations of amphibians, birds, fish [anadromous, brackish, catadromous, or freshwater], mammals, and reptiles, totalling 561,605 genotyped individuals. Every population entry is georeferenced to permit large-scale spatial analyses, opening a variety of opportunities for overlaying microsatellite genetic data with environmental, geographic, or anthropogenic variables. It allows for population diversity and F_ST_ to be directly compared to species and genetic diversity (e.g. heterozygosity and mean number of alleles) through mapping applications.

MacroPopGen exemplifies the importance and usefulness of collating population genetic data by standardizing data from >1000 different studies, allowing for large-scale comparisons and many future applications, including latitudinal analyses, spatial or temporal analyses, taxonomic comparisons and regional assessments of genetic diversity across taxa or in relation to anthropogenic effects. Previous works focusing on older markers have already shown incredible usefulness in testing a variety of genetic and ecological theories^1,2,9^. We provide a baseline database for future works to build from and to compare to, particularly for comparing results to different, newer technologies. We urge future population studies using newer technologies to strive for a similar standardized repository for reporting population-specific statistics.

## Methods

### Data collection

To collect population-genetic data from vertebrate populations located in the Americas, we first scanned Web of Science and Google Scholar for relevant articles using key search terms including country of occurrence, species common names, author names, and scientific names in combination with “microsatellite”, “distinct population”, and/or “F_ST_”. A full list of the 1304 key terms and combinations used can be found online^22^. We also cross-referenced the list of bird microsatellite papers from Willoughby *et al*.^9^.

Search results with over 1000 hits would be filtered where if two consecutive pages did not yield a relevant result, further pages would not be considered (on average the first 15 pages on Google Scholar would be filtered for relevant articles). This preliminary screening limited results down to 6,297 peer-reviewed studies, technical reports, dissertations and government documents, of which only 1,308 fulfilled our criteria, including 142 of which were obtained from Willoughby *et al.’s*^9^ bird reference list. Once a study was selected, we extracted where possible: population locality name, latitude-longitude coordinates, average population-specific F_ST_ (Wright’s F_ST_ or Weir & Cockerham’s unbiased F_ST_ estimator θ_FST_^23,24^), population-specific observed and expected heterozygosity averaged across loci (H_O_/H_E_, respectively), sample size (N), population-specific mean number of alleles per loci (MNA), study-specific corrected allelic richness (AR), and the number of microsatellite loci used in the study. For each population, we also documented the taxonomic group (amphibians, birds, fish [anadromous, brackish, catadromous, or freshwater], mammals, or reptiles), family, genus, species, common name, continent, and country. We chose not to include marine species because microsatellites have typically been unable to detect fine-scale population structure in such species, in contrast to the increased power and resolution of more recent genome-scale analyses for such species^25^. Instead we focus on terrestrial and aquatic ecosystems.

All populations were georeferenced in decimal degrees; if coordinates were not provided, they were inferred from the text or maps in a study. To calculate a metric of population-specific F_ST_, we consulted pairwise F_ST_ tables and averaged across values that included the focal population, or population group if there was no significance between one or more population pairs. When only a global or regional F_ST_ was reported then that value would be used for all populations within the study; such F_ST_ values are indicated in the database where applicable.

### Inclusion criteria and assumptions

A study was retained if two criteria were met: 1) microsatellites were used as molecular markers and 2) genetic differentiation was measured by Weir and Cockerham’s pairwise F_ST_ as opposed to other differentiation estimators because of its wide usage. Microsatellites were favoured over other molecular markers (e.g. SNPs, mitochondrial DNA, allozymes, RAPD, etc.) because their polymorphic nature allows them to resolve population structure at fine scales, particularly for closely related populations^26,27^. Additionally, microsatellites have higher mutation rates than other markers^21,28^ and have been one of the most widely used genetic markers in recent decades^21^. Therefore, microsatellites presently provide an abundance of collectable data across taxa relative to more recent molecular developments associated with single nucleotide polymorphisms (SNPs) or barcoding. While barcoding can assess phylogenetic signals across populations and species, microsatellites allow for the comparison of genetic characteristics between populations such as heterozygosity and allelic diversity, which has been noted to indicate levels of inbreeding or adaptive potential^29–32^.

Studies were assumed to have used selectively neutral nuclear microsatellite loci unless otherwise indicated because microsatellites are located within non-coding regions of the genome^33^ and have relatively fast mutation rates^33,34^. Microsatellite loci are often selected based on their polymorphism due to these faster mutation rates, causing concern that microsatellites may bias measures of genetic diversity compared to whole DNA sequencing-based measures^34,35^. Polymorphism bias has also been recognized in studies using other genetic markers such as SNPs^21,34,36,37^, and will continue to present challenges in genetic studies. An inherent assumption of this database is that ascertainment bias is similar across all studies and taxa, and therefore comparable. Additionally, previous work^9^ has concluded that the number of loci and primer type (whether cross-species or focal species) were not important in explaining variability in genetic diversity, an indication that ascertainment bias may not be very significant for large quantities of microsatellite data such as this database. Regardless, we tested ascertainment bias with a subset of the database, as described below.

### Demarcating Populations

Populations were considered genetically distinct above a threshold F_ST_ value of 0.02. F_ST_ was used as the statistical measure of differentiation because of its standardized and common use in the literature for measuring genetic differentiation. The chosen threshold was based on a previous analytical review^38^, which indicated that genetic differentiation is not negligible if F_ST_ ≥ 0.05, but an F_ST_ value as low as 0.01 can also denote statistically significant differentiation^38^. While lower values of F_ST_ (0.02 to 0.01) are sufficient to show significant genetic differentiation, such values are more relevant for distinguishing specific taxonomic groups, such as marine fish populations which exhibit more gene flow^38,39^. Freshwater and terrestrial species tend to experience lower rates of gene flow than marine species and therefore an F_ST_ threshold above 0.01 is more appropriate^13,39^. To avoid accepting biologically insignificant population differentiation (type I error) or rejecting biologically significant differentiation (type II error) when demarcating populations, we considered the significance of F_ST_ values where available. We ensured that any pairwise comparisons >0.02 were statistically significant; we also checked significance when F_ST_ was <0.02 and significance implied two separate populations despite a lower F_ST_. We also accounted for sample sizes with respect to significant F_ST_. If sample size was five or less (occurring <0.1% of all cases in this study) and populations were found to be significantly different, the populations were instead grouped as one unless an adequate biological explanation was provided (n = 5). Likewise, if sample size was very large (e.g. >50) but F_ST_ was <0.02, consideration would be taken to determine if the populations were significantly different given the statistical support large sample sizes provide (usually given by p-values in the specific study, n = 63 cases where n ≥ 50 but F_ST_ ≤ 0.02). Additionally, if multiple studies were conducted in the same location for the same species, data from the most recent study or the one with the most microsatellite loci was used (n = 268 populations were duplicates and removed). When F_ST_ tables were unclear (e.g. many low F_ST_ values and no significance given), we considered results from population structure analyses (e.g. STRUCTURE, BAPS, etc.) to make informed decisions about population structure.

### Geographic Breadth

We also report (i) how differentiated each population is in relation to all other populations it was compared to by calculating the average F_ST_ between a focal population and all other populations within that study, and (ii) the number of populations included in the calculation as well as the geographic distance or breadth that they span. For example, low F_ST_ values resulting from only a few sampling locations (e.g. 5) in a small geographic region (e.g. 10 km) may have a different interpretation than low F_ST_ values across many (e.g. >10) sampling locations in a broad geographic range (e.g. 10,000 km). To estimate the geographic breadth that sampled populations cover, we obtained coordinates for each population including locations that had been combined into one population. These data were put into a separate file that contains 10,921 sampling localities. Next, we used custom code^22^ utilizing the R package geosphere to calculate the maximum, minimum, and mean distances in metres between all populations of a study; distances are reported in metres in the database. We additionally note how many sampling localities make up each population in the database and how coordinates were obtained/estimated for populations that encompass multiple sampling localities.

### Statistical Analysis

To calculate mean genetic diversity for taxonomic groups and continental regions we used generalized linear mixed models (GLMMs) that accounted for the random effect of study, species, genus, and family. Fixed effects included either the taxonomic group, or the continental region. Beta distributions were used to model H_O_ and F_ST_ (R package glmmTMB v 0.2.2.0) because both these response variables and distributions are bounded between zero and one with no exact zeros or ones. Gamma distributions were used for MNA (R package lme4 v 1.1-18-1) as MNA follows a positively right skewed distribution characteristic of gamma distributions. We then used the R package and function emmeans (v 1.2.3) to calculate the mean values while accounting for model structure. For the models that used beta distributions, we used the function back.emmeans (R package RVAideMemoire v0.9-69-3) to back transform estimates.

To compare the degree of variation in each taxonomic or continental group, we calculated the coefficient of variation grouped at the species level for H_O_, MNA, and F_ST_. Mixed models using the gamma distribution and random effects of reference, genus, and family were constructed. We then used model selection to see which between taxonomic group or continental region best explained differences between groups.

We assessed trends of ascertainment bias related to microsatellite loci development using a subset focusing on North American mammalian data (n = 1579 populations, 73 species)^22^. In addition to the number of microsatellite loci, we obtained from 230 mammalian studies the number of species used to develop those loci (ranged from 1 to 7), and whether the species were focal (n = 384), non-focal (n = 545), or mixed (n = 692), as well as information on the senior author’s country of affiliation. Using IUCN descriptions for each species, we also determined whether the species was harvested and to what extent (no n = 317, low n = 957, or high n = 347), the species’ IUCN status (Least Concern n = 1335, Near Threatened n = 45, Vulnerable n = 193, Endangered n = 41, Critically Endangered n = 7), whether the species was of conservation concern (no n = 561, low n = 211, or high n = 849), charismatic (no n = 495, low n = 189, or high n = 937), or of economic value (no n = 602, low n = 887, or high n = 132). Extent of harvesting was determined by the degree of harvesting described in IUCN’s “Use and Trade” category: none (“no”), subsistence or local harvesting (“low”), or substantial commercial harvesting (“high”). Conservation concern was specified to account for species that may have a lower IUCN rank (e.g. Least Concern, LC) but still have populations at risk or aspects of their habitat at risk (e.g. 563 LC species were still of conservation concern and therefore considered as “low”); this was largely described in IUCN’s “Threats” and “Conservation Action” categories. Charisma of species was somewhat subjective as it was determined by how generally well-known the species was, and whether the species may be considered a nuisance which would negatively affect their charisma score (e.g. the coyote is well known but can be considered a pest and as such its score was “low”). Economic value of a species was determined by the “Use and Trade” section, where if the species was commercially harvested it would be considered to have economic value (“high”); if the harvest has declined or is relatively low, a species’ economic value was considered as “low”.

We tested the fixed effects and interactions among these factors for ascertainment bias as well as the random effects of reference, species, genus, and family. We used GLMMs, using a beta distribution for H_O_ (R package glmmTMB) and a gamma distribution for MNA (R package lme4). Following Zuur *et al*.^40^ guidelines for forwards and backwards model selection, we used the likelihood ratio test to find significant factors for the H_O_ and MNA models, respectively.

### Code and Data Availability

The data and R code used for the analyses are available from FigShare^22^.

## Data Records

Data from the MacroPopGen database is hosted at Figshare^22^ and can be downloaded as one XLSX file. It consists of 9,098 rows (distinct populations), and 24 columns. The columns include taxonomic identifiers (family, genus, species, common name), population locality information, and study-specific data (sample size, population-specific F_ST_, observed and expected heterozygosity, mean number of alleles, standardized allelic richness, latitude and longitude coordinates, reference ID, and year).

An additional XLSX file containing the corresponding references for each reference ID, and the list of key terms used in searches is also available on Figshare^22^. Most of the references were published in English, although a minority are in Spanish.

## Technical Validation

### Geographic and taxonomic bias

Between 1994 and 2017, most population microsatellite data came from species studied in North America (85.1%, Table 1). Fish species were the most represented taxonomic group, making up 44.8% of the database (Table 1). Salmonid species made up 55.9% of fish population data and represented 25.0% of data across all taxa.

**Table 1:** Summary statistics for data collected from microsatellite studies published between 1994 and 2017 broken down by taxonomic group.

	Amph	Bird	Anad	FW	Mam	Rep	NOR	CEN	CAR	SOU	Total
***Unique families***	17	66	6	42	37	28	135	31	16	98	**195**
***Unique genera***	46	170	9	99	93	66	308	40	18	173	**480**
***Unique species***	104	254	15	231	158	133	578	45	26	282	**897**
***Number populations***	1117	608	1315	2704	1943	1349	7738	230	107	1015	**9090**
***Studies***	136	265	72	298	344	203	962	46	32	299	**1308**
***Countries***	10	28	2	16	19	30	4	6	15	14	**39**
***Published year range***	2001–2016	1997–2017	1997–2016	1997–2017	1994–2016	1997–2017	1994–2017	2002–2016	2002–2017	1997–2017	**1994–2017**
***Mean latitude***	32.713	25.923	50.546	37.445	34.188	27.520	43.415	11.643	18.384	−14.585	**35.83**
***Total number of loci***	10870	6713	18958	28069	23213	13869	88259	2421	1050	10701	**102431**
***Mean number loci per study***	9.740	10.987	14.439	10.450	11.947	10.273	11.437	10.526	9.813	10.543	**11.29**
***SD number loci across studies***	3.689	6.711	4.0329	4.465	5.587	4.928	5.161	5.124	6.924	3.975	**5.08**
***Total individuals genotyped***	46015	48393	181606	140569	91147	50978	507765	8990	3904	40946	**561605**
***Median study N***	22	34	83	30	25	22	30	28	20	24	**30**
***SD N***	88.472	126.508	174.205	198.611	96.694	69.460	156.897	54.052	35.703	71.330	**147.43**
***Mean H***_***O***_	0.596*	0.592*	0.627*	0.566*	0.594*	0.582*	0.596*	0.610*	0.576*	0.567*	**0.59**
***SE H***_***O***_	0.023*	0.031*	0.014*	0.077*	0.017*	0.019*	0.022*	0.029*	0.009*	0.012*	**0.16**
***Mean MNA***	5.650*	5.339*	7.807*	5.629*	4.855*	6.077*	4.838*	5.536*	4.110*	5.203*	**7.92**
***SE MNA***	0.313*	0.189*	0.692*	0.219*	0.140*	0.293*	0.159*	0.383*	0.348*	0.212*	**5.57**
***Mean population F***_***ST***_	0.106*	0.052*	0.062*	0.092*	0.091*	0.086*	0.073*	0.120*	0.079*	0.086*	**0.13**
***SE population F***_***ST***_	0.015*	0.006*	0.011*	0.009*	0.009*	0.009*	0.009*	0.017*	0.005*	0.008*	**0.12**

N = sample size; H_O_ = observed heterozygosity; MNA = mean number of alleles; SD = standard deviation; SE = standard error. Amph = amphibians; Anad = anadromous fishes; FW = freshwater fishes; Mam = mammals; Rep = reptiles; NOR = North America; CEN = Central America; CAR = Caribbean; SOU = South America. Brackish and catadromous fishes are not shown due to their low number of populations (25 and 33, respectively).

^*^Calculated to account for model structure. See text for details.

**Fig. 1:**
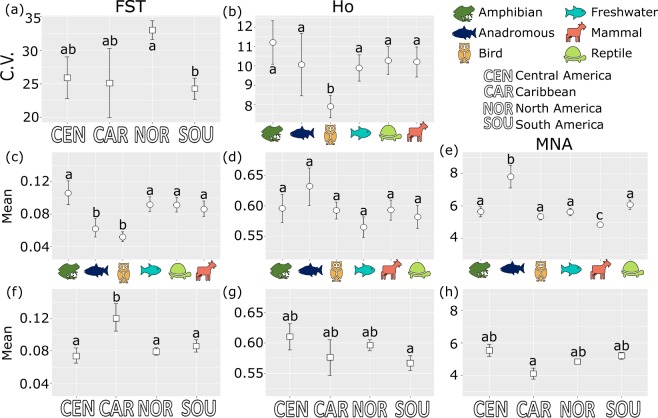
Coefficient of variation and mean values for observed heterozygosity (H_O_), mean number of alleles (MNA), and population-specific F_ST_ calculated to account for GLMM structure. Error bars represent standard error. Significant differences between groups indicated by letter grouping where groups sharing the same letter(s) are not significantly different from one another. (**a**,**b**) Coefficient of variation calculated across (**a**) taxonomic groups (circles) and (**b**) between continental regions (squares). (**c**–**e**) Mean (**c**) F_ST_, (**d**) Ho, and (**e**) MNA calculated across taxonomic groups. (**f**–**h**) Mean (**f**) F_ST_, (**g**) Ho, and (**h**) MNA calculated between continental regions.

When accounting for model structure, mean population genetic diversity differed significantly between some continental regions for H_O_ and MNA (Fig. 1). Populations of South American species had the lowest H_O_ while Caribbean populations showed significantly lower MNA (Table 1, Fig. 1). Despite some significant differences, the range of mean population genetic diversity metrics among continental regions was limited, between 0.57 and 0.61 for H_O,_ and 4.11 and 5.5 for MNA (Fig. 1). Continental population differences in F_ST_ were stronger than for genetic diversity metrics, wherein Caribbean populations showed significantly higher population-specific F_ST_, suggestive of less gene flow overall for these populations. This result follows general island-mainland expectations where island populations tend to be more isolated than mainland populations^41,42^.

Among taxonomic groups, populations of anadromous fish had statistically higher mean genetic diversity (MNA = 7.8), and lower average F_ST_ values (0.06) aside from birds (mean F_ST_ = 0.05) (Fig. 1), consistent with previous work^12,13^. Mammalian populations also had lower mean MNA than all other groups (Fig. 1). However, there were no significant differences in mean H_O_ between taxonomic groups (Fig. 1).

### Variation among taxonomic and continental groups

**Fig. 2:**
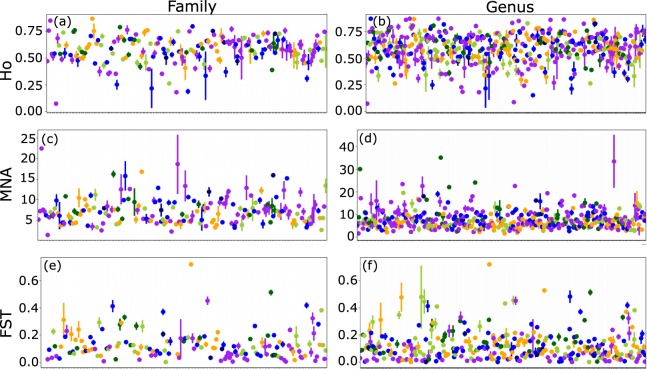
Microsatellite observed heterozygosity (H_O_), mean number of alleles (MNA), and population-specific F_ST_ averaged across each vertebrate group. Colours indicate the taxonomic group each family or genus belongs to: dark green = amphibians, purple = birds, blue = fish, orange = mammals, light green = reptiles. Error bars represent standard error. (**a**,**c**,**e**) Ho, MNA, and F_ST_ are averaged across vertebrate families (n = 195). (**b,d**,**f**) Ho, MNA, and F_ST_ are averaged across vertebrate genera (n = 480).

There were significant differences in the coefficient of variation for H_O_ among taxonomic groups but not continental regions, with bird species showing the least variation (Fig. 1). There were no significant differences in the coefficient of variation for species MNA across taxonomic groups or continental regions (Fig. 1). For F_ST_, the only statistical difference was for the coefficient of variation to be larger in North American species relative to species in other regions, i.e. no taxonomic group differences in F_ST_ variance were found (Fig. 1). More variance among taxonomic distinctions was observed when considering within-family and within-genera variance in genetic metrics (Fig. 2). For example, the mean family H_O_ ranged between 0.07–0.88, while MNA ranged from 1.40–24.97, and mean F_ST_ ranged from 0.0008–0.72; genera averages had a similar range for both metrics.

### Bias with microsatellite loci

**Fig. 3:**
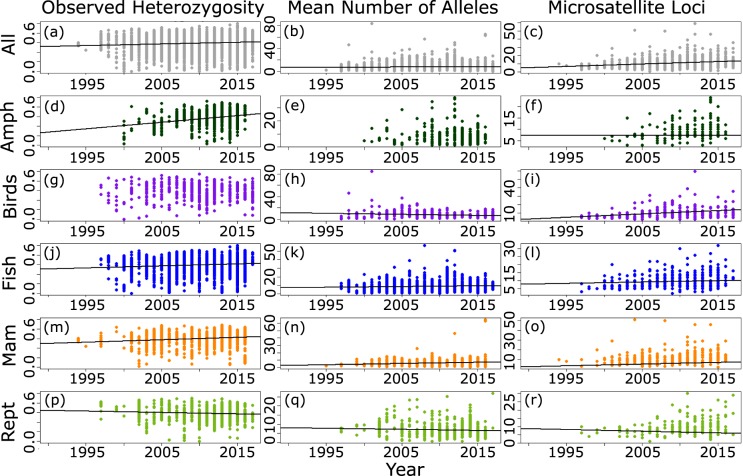
Observed heterozygosity, mean number of alleles, and number of microsatellite loci for populations of each taxonomic group sampled between the years 1994 to 2017. (**a–c**) All vertebrate groups together; (**d–f**) only amphibian species; (**g–i**) bird species; (**j–l**) all fish species; (**m–o**) mammalian species; (**p–r**) reptile species. Linear models are indicated for significant relationships.

**Fig. 4:**
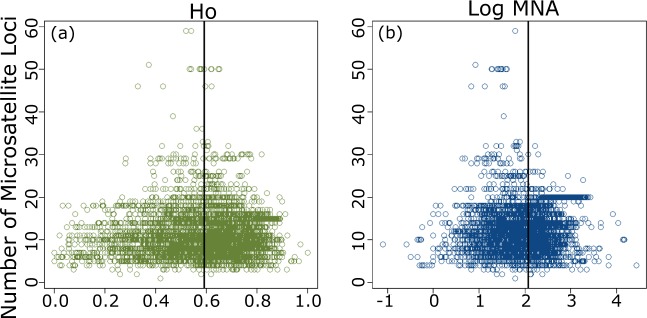
Funnel plots for all populations; y axis for both plots is the number of microsatellite loci, and (**a**) x axis is observed heterozygosity (H_O_) or (**b**) mean number of alleles (MNA). Vertical line represents the mean value.

We assessed how genetic diversity and the number of microsatellite loci employed in empirical research has changed over time using linear models (Fig. 3). There has been a significant trend for increasing number of loci per year (R^2^ = 0.07, p <0.001) as well as a weak increase in genetic diversity with year (H_O_: R^2^ = 0. 0.009, p <0.001 and MNA: R^2^ = 0.001, p = 0.003). Additionally, we evaluated bias with respect to the number of microsatellite loci and the degree of genetic variation in H_O_ and MNA using funnel plots (Fig. 4) and linear models. The plots appear to be largely symmetrical and show little bias with respect to number of loci, indicating the data capture a reasonable degree of genetic variation for the number of loci used. Note that we could not use a formal funnel plot test such as the Egger test because we do not have variance for H_O_ and MNA for each study. However, the number of microsatellite loci was a significant predictor in linear models for both H_O_ and MNA (p <0.001 for both), although adjusted R^2^ values were very small (0.002 and 0.03, respectively).

### Ascertainment bias

After model selection testing for ascertainment bias with respect to loci type and origin, only the interaction between level of harvesting and conservation concern as well as the random effects of reference, family, and genus were significant for the H_O_ model (Table 2). For the MNA model, the significant factors only included the interaction between conservation concern and charisma, as well as the random effects for reference and genus. None of the factors associated with microsatellite bias were retained in model selection (i.e. number of species used to derive loci, whether those species were focal, non-focal, or mixed). These results are consistent with previous assessments^9^ but indicate that microsatellite loci and loci origin do not significantly affect genetic diversity metrics when analyzed across diverse taxa.

**Table 2:** Summary of model selection results for testing ascertainment bias within H_O_ and MNA.

Model	AIC	DF
H_O_ ~ 1 + (1|Reference) + (1|Species) + (1|Genus) + (1|Family)	−2196.0	6
H_O_ ~ MsatType + (1|Reference) + (1|Genus) + (1|Family)	−2183.6	7
H_O_ ~ ConservC + (1|Reference) + (1|Genus) + (1|Family)	−2202.3	7
H_O_ ~ Harvested + ConservC + (1|Reference) + (1|Genus) + (1|Family)	−2212.2	9
H_O_ ~ MsatType + ConservC + (1|Reference) + (1|Genus) + (1|Family)	−2198.2	9
H_O_ ~ Harvested * ConservC + (1|Reference) + (1|Genus) + (1|Family)	−2215.2	13
H_O_ ~ MsatType + Harvested * ConservC + (1|Reference) + (1|Genus) + (1|Family)	−2214.2	14
H_O_ ~ NSpp + Harvested * ConservC + (1|Reference) + (1|Genus) + (1|Family)	−2215.3	14
H_O_ ~ msat + Harvested * ConservC + (1|Reference) + (1|Genus) + (1|Family)	−2212.1	15
MNA ~ ConservC:Charisma + (1|Reference) + (1|Genus) + (1|Species)	4015.6	13
MNA ~ NSpp + ConservC: Charisma + (1|Reference) + (1|Genus) + (1|Species)	4016.2	14
MNA ~ NSpp + MsatLoci + ConservC + AuthorCountry + ConservC: Charisma + (1|Reference) + (1|Genus) + (1|Species)	4021.6	19
MNA ~ NSpp + MsatLoci + MsatType + Harvested + ConservC + Economic + Charisma + AuthorCountry + ConservC: Charisma + (1|Reference) + (1|Genus) + (1|Species)	4031.5	25
MNA ~ NSpp + MsatLoci + MsatType + Harvested + ConservC + Economic + Charisma + AuthorCountry + NSpp: MsatLoci + NSpp: MsatType + MsatLoci: MsatType + Harvested: ConservC + Harvested:cmn + ConservC: Charisma + (1|Reference) + (1|Genus) + (1|Species)	4043.7	36
MNA ~ NSpp + MsatLoci + MsatType + Harvested + ConservC + Economic + Charisma + AuthorCountry + NSpp: MsatLoci + NSpp: MsatType + MsatLoci: MsatType + Harvested: ConservC + Harvested: Charisma + ConservC: Charisma + (1|Reference) + (1|Species) + (1|Genus)	4050.9	37

NSpp: number of species used to derive loci; MsatLoci: total number of microsatellite loci; MsatType: microsatellite type (focal, non-native, native); Harvested: level of harvesting; ConservC: degree of conservation concern; Economic: economic value; Charisma: charisma of focal species; AuthorCountry: senior author’s country of residence.

## ISA-Tab metadata file

Download metadata file
